# Sulfuryl Fluoride Poisonings in Structural Fumigation, a Highly Regulated Industry—Potential Causes and Solutions

**DOI:** 10.3390/ijerph16112026

**Published:** 2019-06-06

**Authors:** Tracy Barreau, Sumi Hoshiko, Rick Kreutzer, Svetlana Smorodinsky, John Talarico

**Affiliations:** 1 California Department of Public Health, Environmental Health Investigations Branch, Richmond, CA 94804, USA; tracy.barreau@cdph.ca.gov; 2 California Department of Public Health, Center for Healthy Communities, Richmond, CA 94804, USA; svetlana.smorodinsky@cdph.ca.gov

**Keywords:** fumigation, sulfuryl fluoride, chloropicrin, Vikane, pest control, extermination, termites, pesticide, pesticide illness surveillance

## Abstract

Structural fumigations using sulfuryl fluoride for the extermination of dry-wood termites are conducted by the thousands in California and other warm-weather states. Sulfuryl fluoride is an odorless gas that targets the nervous system and can cause respiratory irritation, pulmonary edema, nausea, vomiting, seizures, and death. Structural voids or compartments such as wall sockets, crawl spaces, cabinets, or cells in air mattresses may create ongoing exposure after a structure has been certified as safe. The authors describe a case of potential sulfuryl fluoride exposure to a family following home fumigation. Despite regulation, sulfuryl fluoride poisonings from structural fumigations continue to occur. This article examines the physical characteristics of sulfuryl fluoride and the regulatory oversight of its application, in an effort to understand how and why these poisonings happen. Increasing aeration times of fumigated structures, overseeing monitoring efficacy, and using technology to capture clearance data could reduce sulfuryl fluoride exposure and illness.

## 1. Introduction

Structural fumigations for termite extermination are conducted by the thousands annually in the United States. The active chemical commonly used is sulfuryl fluoride, an odorless gas that targets the nervous system. In this article, we discuss a case of potential sulfuryl fluoride exposure to a family following home fumigation and examine the oversight process in an effort to understand how to reduce exposure risk.

## 2. Case Study

In early March 2016, an emergency duty officer of the California Department of Public Health (CDPH) responded to a call from a family describing symptoms suggestive of chemical poisoning following home fumigation to eradicate dry-wood termites [1]. The fumigation occurred on February 17, 2016, and the house was cleared for reentry after a standard length of time by the structural pest control operator (SPCO) on February 19, 2016, with no reported detection of sulfuryl fluoride at the 1 part per million (ppm) clearance level [2]. Structural fumigations are conducted using sulfuryl fluoride, a colorless, odorless, lethal gas, which is heavier than air [3,4]. Sulfuryl fluoride is regulated, and it cannot be purchased over the counter for use by the general public. Chloropicrin, another toxic pesticide, is used as a warning agent due to its sharp irritating odor and property as a strong lachrymator (Table 1) [3]. Common practice is to place a pan containing several ounces of chloropicrin on the floor in the structure to be fumigated; in this case study, a pan of chloropicrin was placed on the floor of the garage. 

The parents of a family with four children (9-year-old twins, a 5 year old, and a 13-month-old child) reported a strong chemical odor and the presence of a white residue covering furniture and floors upon entering the house after it had been certified safe for reentry, per the father’s description in a phone call with CDPH staff. The family contacted the SPCO and the County Agricultural Commissioner, who oversees fumigations, and proceeded to open windows, drawers, cabinets, and sliding doors. They reported burning and watering eyes, itchy skin, headaches, a feeling of lungs hurting, fatigue, dry and burning throats, coughing, extreme thirst, and unusual head-banging behavior by the toddler. They left and stayed in a hotel that night. In response to the complaint, the SPCO returned the next day to place fans in the residence. Fumigant testing by the SPCO is described in the following section. 

The parents returned over the following days to remove the residue from the furniture. They reported developing skin irritation and rash, dizziness, nausea, chest pain, and symptoms that include burning throat or headache, which could persist for hours. They described reactions to fumes emanating from bags of belongings that had been sealed prior to fumigation. The property manager who was present the day of reentry reported difficulty breathing, and neighbors reported a chemical odor when entering the house. Several weeks later, one of the children developed a rash after wearing a sports uniform that had been in the house. The father returned to the house three weeks after clearance to pack up scuba equipment. Upon releasing stored air from a buoyancy compensator device, he immediately developed a piercing headache and began vomiting [2]. A Pesticide-Related Illness Report filed in early March after evaluation by a family physician noted chemical exposure and recommended avoidance of further exposure. In follow-up by CDPH several months later, the family mentioned that their son, who was 13 months of age and beginning to verbalize at the time of the incident, had stopped using words in the three to four months following the incident. The family ultimately never returned to live in the house.

## 3. Fumigant Testing and Follow-Up Investigations

According to the SPCO, the California Aeration Plan, which included set up of aeration ducting, 12 hours of aeration, and clearance testing, was followed [5]. Although not present at the time of application, the County Agricultural Commission conducted a partial inspection later that day, which consisted of walking around the property and noting posted warning signs, condition of tarps, and the fumigation seal. Testing by the SPCO on the day of clearance (in the morning and after the house was reentered by the resident) did not detect sulfuryl fluoride using a direct reading instrument with sensitivity at 1 ppm [2]. As mentioned, the following day (after clearance) the SPCO installed large fans. Three days following clearance, sulfuryl fluoride testing was again performed by the SPCO, and again no sulfuryl fluoride was detected. Chloropicrin was not tested. According to the family, sulfuryl fluoride measurements were taken while aeration fans were blowing and the windows were open [2]. The SPCO tested for chloropicrin four days after clearance, without detection, using a method with a detection limit of 100 ppb (parts per billion). After the chloropicrin testing, fans were removed, and the house was closed again. A follow-up investigation initiated in early March, consisting primarily of interviews by the County Agricultural Commissioner’s office did not identify any violations [2]. There are no requirements for the SPCO to document measurements taken to certify the house safe for reentry. So, no data logs are available that would specify locations, times, and concentrations to verify any measurements; only a summary statement from the SPCO in response to the complaint, that notes the non-detect results of sulfuryl fluoride testing on February 19, February 22, and February 24 (a safe in the garage was tested) and chloropicrin on February 23.

As part of the Agricultural Commissioner’s investigation, swab sampling was performed on the outside of a fumigation bag that reportedly had triggered itchiness and a skin rash when the father reached in to obtain medicines. However, the lab’s multi-residue screen list did not include fluoride, the breakdown product of sulfuryl fluoride, so if any residues of sulfuryl fluoride were remaining, they would not have been detected [2]. It is commonly stated that sulfuryl fluoride used in structural fumigation does not leave a residue; however, these claims are based on limited studies. Sulfuryl fluoride used in food fumigation leaves fluoride residues, which are regulated due to toxicity concerns, especially for children [6,7].

On March 28, over five weeks after fumigation, the California Department of Pesticide Regulation (CDPR) conducted post-fumigation monitoring of the residence indoor air for chloropicrin and sulfuryl fluoride [8]. Chloropicrin was not detected at any of the five locations tested and sulfuryl fluoride was not detected in 11 breathing zone samples. However, the bed in the master bedroom had an air bladder as part of the mattress, and air exhausted from two air cells in the bed contained sulfuryl fluoride at 2.4 ppm, over twice the clearance level, despite the length of time that had elapsed since fumigation [8]. The Agricultural Commissioner’s Pesticide Investigation Report concluded that it was unclear if the symptoms experienced by the family were caused by the fumigant [2].

## 4. Discussion

**Figure 1:**
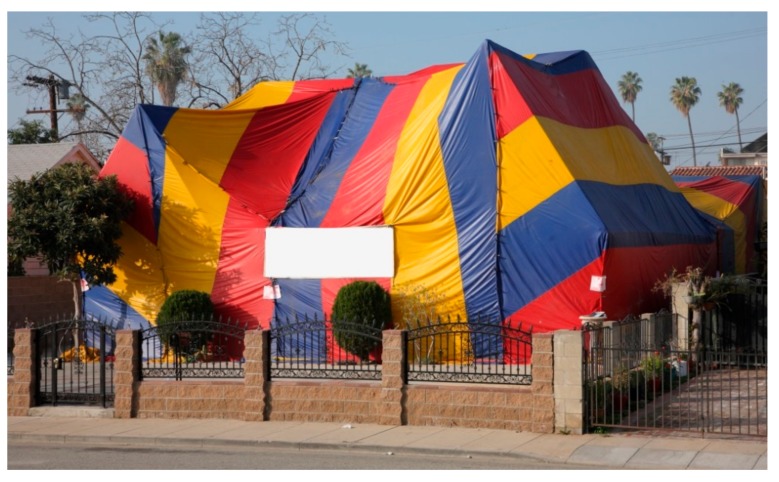
House tented for termite fumigation.

Structural fumigation is a pest control method in which sulfuryl fluoride gas is pumped into a structure that has been enclosed with tarpaulins, commonly known as “tenting” (Figure 1) [9,10]. During fumigation, concentrations of sulfuryl fluoride in single-family residences are estimated to range from 1400 to 3800 ppm [4], before the structure is aerated to the target sulfuryl fluoride clearance level of 1 ppm. At the time of this report, there are no requirements to measure chloropicrin, the warning agent, as part of the clearance procedures.

Between 2003 and 2014 (the most recent year for which data are available), 59 reported incidents of sulfuryl fluoride-related illnesses following structural fumigations in California received follow-up investigation, according to surveillance data in the Pesticide Illness Surveillance Program (PISP), maintained by the CDPR [11]. This database may include medical reports submitted by physicians, review of worker compensation reports, illness investigation reports from complaints to the County Agricultural Commissioner’s offices, and medical reports collected from local health officers. The CDPR counted 86 individuals among the 59 incidents, although the number of persons may have been as many as 100, as in some instances, additional persons were mentioned in the reports as having symptoms, but information was less complete, (e.g., the person may not have participated in a CDPR interview or sought medical care) so these individuals are not included in the CDPR’s count (Table 2) [11]. In 7 of 59 incidents (12%), or 7 of 86 individuals (8%), the exposure resulted in a fatality. Over half of the incidents, but none of the fatalities, involved persons who were exposed after the structure had been cleared [11]. 

Measurements taken during follow-up investigation usually failed to detect sulfuryl fluoride, but in some cases exposures were found post-clearance. For example, in one incident described in PISP summary data, “A utility technician experienced symptoms after turning on the gas at a recently fumigated home. Tests revealed levels between 0 and 4 ppm sulfuryl fluoride 12 days after the SPCO cleared the home. The residents smelled an odor and reported similar symptoms.” In another instance, a couple experienced symptoms upon returning to their recently fumigated home. The husband sought care a month later for persistent symptoms, and follow-up testing detected sulfuryl fluoride at 1 ppm a month following fumigation. Similar to the case study in this report, another incident also involved exposure from a mattress: “The couple slept in their bed that night... The SPCO tested the apartment and found the mattress to be off-gassing the fumigant.” Three incidents involved exposures occurring at banks. In addition to the fumigant being retained in the bank vault itself (in one instance at 20 ppm), according to the notes for one incident, “Possible sources for the rising levels may have been sulfuryl fluoride seeping out of locked drawers and cabinets that were not aerated.” See Table 2 for examples of incidents in the PISP surveillance data [11]. 

Symptoms generally appear to resolve quickly in non-fatal cases. But, health consequences of acute sulfuryl fluoride exposure may be long lasting, such as a Florida 2015 case, in which a 9-year-old boy suffered permanent brain damage from sulfuryl fluoride exposure after his family’s home was fumigated [12].

**Figure 2:**
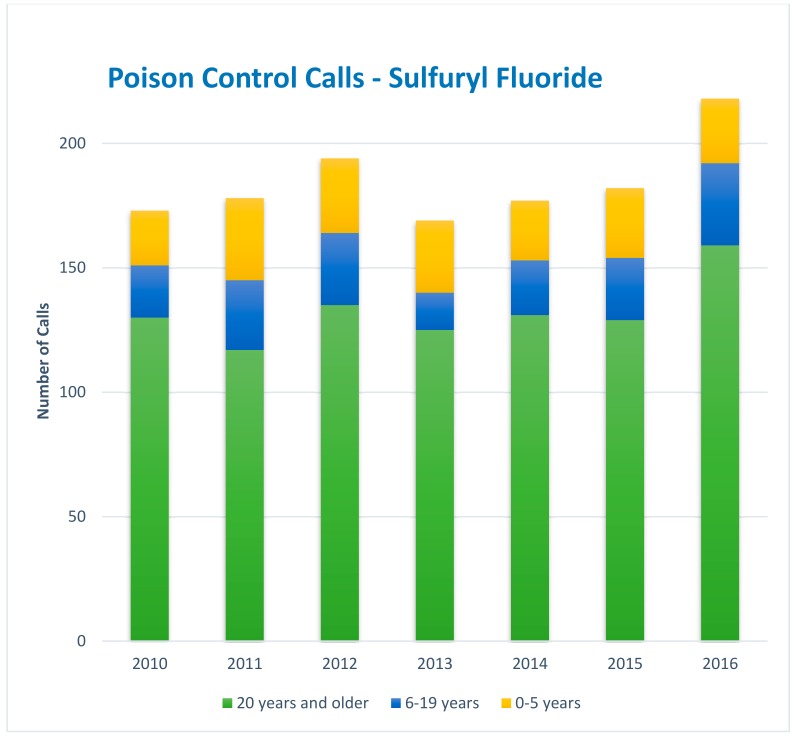
Calls to California Poison Control concerning sulfuryl fluoride exposure 2010–2016.

In addition to the cases described above that are captured by the CDPR’s Pesticide Illness Surveillance Program, additional cases involving the public’s exposures to sulfuryl fluoride/chloropicrin combinations during structural fumigation can be found in calls to the California Poison Control System. A query of the Poison Control database identified a total of 1291 calls concerning sulfuryl fluoride/chloropicrin between 2010 and 2016 (not including animal exposures or information-only queries) (Figure 2) [13]. All calls are evaluated to confirm exposure to sulfuryl fluoride/chloropicrin and determine what if any health effects may be anticipated or may have occurred. The evaluation process may conclude that the exposure would not have had an effect or would be non-toxic, or the health effect experienced may be deemed unrelated to exposure. During this time, 779 (65%) of the queries were determined to have had some health effect, mostly in cases reporting irritation which is assumed to be secondary to exposure to chloropicrin, the warning agent (excluding 31 cases that could not be followed). If the situation is one that (1) involves exposure to a specific pesticide, (2) symptoms are experienced, and (3) health care is sought, the case is referred to the CDPR for follow-up investigation. A substantial proportion of the sulfuryl fluoride/chloropicrin queries to Poison Control involved children; Figure 2 shows Poison Control calls regarding sulfuryl fluoride/chloropicrin exposures to very young children aged 0–5, children aged 6–19, and adults 20 years and older, with roughly 30% of calls concerning children aged 0–19 [13]. 

## 5. Sulfuryl Fluoride Health Effects and Exposure Guideline Levels 

Sulfuryl fluoride is a toxic gas that targets the central nervous system [14,15]. It is rapidly absorbed when inhaled and breaks down into several components that travel through the bloodstream, reaching the lungs, kidney, spleen, nasal tissues, and brain [14,15]. Exposure may result in respiratory irritation, shortness of breath, pulmonary edema, nausea, vomiting, stomach pain, itching, central nervous system depression, slowed gait, slurred speech, extremity numbness, muscle twitching, seizures, and death due to respiratory failure (Table 1) [3,4]. Individuals with a history of chronic respiratory disease are at increased risk. Epidemiological studies showed that fumigation workers using sulfuryl fluoride exhibited neurological effects, including reduced performance on cognitive and memory tests and diminished olfactory function [3]. 

In the case study described here, the health symptoms experienced by the family were consistent with sulfuryl fluoride exposure although they reported experiencing some symptoms that are more specific to chloropicrin exposure, the warning agent, which causes burning eyes and has an odor. Many symptoms are associated with exposure to both chemicals (e.g., nose, throat, and respiratory irritation, and coughing) (Table 1). The relationship between exposure and the reported symptoms of the 13-month-old’s head-banging behavior and possible change in speech development is unclear. Infants and children are generally more susceptible to the toxic effects of pesticides than adults because their brain and nervous systems are still developing and because their immature systems cannot remove pesticides as efficiently as an adult [16]. Sulfuryl fluoride is 3.5 times heavier than air, so it would be found closer to the ground or floor, which may present higher exposures for a child whose breathing zone is closer to the ground than an adult. 

To understand further the anticipated health effects of sulfuryl fluoride at different exposure levels, we can consider the Acute Exposure Guideline Levels (AEGLs), established through a federal advisory committee [17]. These guidelines are used worldwide to provide emergency responders critical information on adverse health effects and threshold limits for once-in-a lifetime short-term exposures to airborne concentrations of acutely toxic chemicals [17]. AEGL-1 is the airborne concentration of a substance above which it is predicted that the general public could experience notable discomfort, but with reversible effects; the AEGL-2 level could confer irreversible or serious, long-lasting effects; AEGL-3 is a level at which the general population could experience life-threatening health effects or death. It is recognized that susceptible individuals could experience effects described at concentrations below the corresponding AEGL. No AEGL-1 values are calculated for sulfuryl fluoride due to inadequate data. The 10 minute AEGL-2 for sulfuryl fluoride is 27 ppm, and the 8 hour AEGL-2 exposure concentration is 6.7 ppm. The 10 minute AEGL-3 concentration (lethal) is 81 ppm and the 8 hour level is 20 ppm [17]. 

Another set of guidelines that are derived to be protective of public health are reference concentrations (RfCs). RfCs are estimates of chemical concentrations in air that are not likely to cause an appreciable risk of noncancer adverse health effects to the public (including sensitive subgroups) for fixed durations of exposure [18]. RfCs are not enforceable standards but are typically used in the risk assessment process for regulatory decision making, with a goal of keeping exposures below RfCs. The risk assessment process also informs rulemaking and standard setting [19]. 

In 2017, the CDPR proposed acute (1 day), short-term (1–2 weeks), and chronic RfCs for residential bystander exposures to sulfuryl fluoride, at 0.41, 0.015, and 0.015 ppm, respectively (short term and chronic have the same level) [20]. The proposed RfCs are below the 1 ppm clearance level deemed safe for reentry and below the range of detection of direct reading instruments used for clearance testing. This would suggest that the clearance standards for residential fumigations treatments allow higher concentrations of sulfuryl fluoride than would be considered protective against noncancer health effects for the general public. 

## 6. Fate and Behavior of Sulfuryl Fluoride

When released into the atmosphere, sulfuryl fluoride has a lifetime of 36 years, eight times longer than previously understood [21]. Furthermore, it may have the potential to be a significant contributor to greenhouse gas effects, as it is increasing in the atmosphere by 5% a year and is 4800 times more potent than carbon dioxide.

Information provided to homeowners and businesses planning to have a structure fumigated indicates that sulfuryl fluoride dissipates quickly [22]. However, multiple cases have occurred where symptoms and odors are reported after a building was aerated for the required time period [11,23]. The fate and behavior of sulfuryl fluoride demonstrated in the laboratory and in actual field applications might play a role in why these poisonings continue to occur.

A study of sorption and desorption of sulfuryl fluoride on different inorganic materials commonly found in households suggests several potential pathways for continuing indoor air exposure after clearance. In a chamber study, sulfuryl fluoride was measured desorbing from different household materials, such as polyester fibers and polystyrene, for up to 40 days [24]. 

In the field, sorption and desorption of sulfuryl fluoride on household materials occurs at different rates [24,25], resulting in prolonged releases of sulfuryl fluoride into indoor air. Structural voids and enclosed spaces (i.e., wall voids, wall sockets, light switches, crawl spaces, attics, cabinets, etc.) can act as reservoirs to trap gas [23,25]. Sulfuryl fluoride can diffuse from these spaces as well as from some commodities, such as rubber membrane air cells, air cells in beds [8], and other compartments [23,25]. These mechanisms provide an opportunity for ongoing exposure after a structure has been certified as safe. 

In a study conducted by Dow, sulfuryl fluoride concentrations were shown to rebound post-certification. The authors measured sulfuryl fluoride concentrations in 15 homes (seven in California and eight in Florida) at different times for up to 48 hours following clearance for reentry [26]. Following a 6 hour aeration period, sulfuryl fluoride concentrations were measured using Miran infrared analyzers and sorbent tubes. Sorbent tube data were then used to calculate 24 hour time-weighted averages (TWA) (average of three, 8 hour samples) for comparison to 5 ppm, the clearance level at the time. TWAs are typically used for smoothing data, but TWAs bias concentrations downwards and mask acute concentration peaks, which are important when evaluating health impacts from a respiratory irritant and nervous system depressant. For example, the 8 hour TWA of 0.26 ppm was reported 16 hours after clearance testing in a Florida home, but the corresponding data using direct readings from the Miran instrument showed a level of 1.9 ppm (~7 times higher than the TWA) in a bedroom [26]. The California data showed measurable concentrations of sulfuryl fluoride in all seven homes studied, for 48 hours after aeration and clearance. While sulfuryl fluoride concentrations generally decreased over time, with the lowest concentrations measured at 48 hours, there were instances when concentrations increased in a later sampling interval, demonstrating the variability in sulfuryl fluoride dissipation [26]. The authors note the variability in dissipation among the homes is likely due to the tightness of the building and desorption of sulfuryl fluoride from permeable items and void spaces within the home. The CDPR’s review of the Dow study concluded, “The data from the Shurdut study (1995) indicate that indoor air concentrations do not go to zero in 24 hours. Thus, there is the likelihood of short-term (seven days or less) as well as acute exposure to indoor air concentrations of sulfuryl fluoride” [14]. 

The Dow studies demonstrate sorption and desorption of sulfuryl fluoride on various media, and the potential for void spaces to be reservoirs, all of which may be contributing to a rebound of sulfuryl fluoride levels after required aeration times and clearance levels are achieved [24,25,26]. 

## 7. Regulatory Oversight

**Figure 3:**
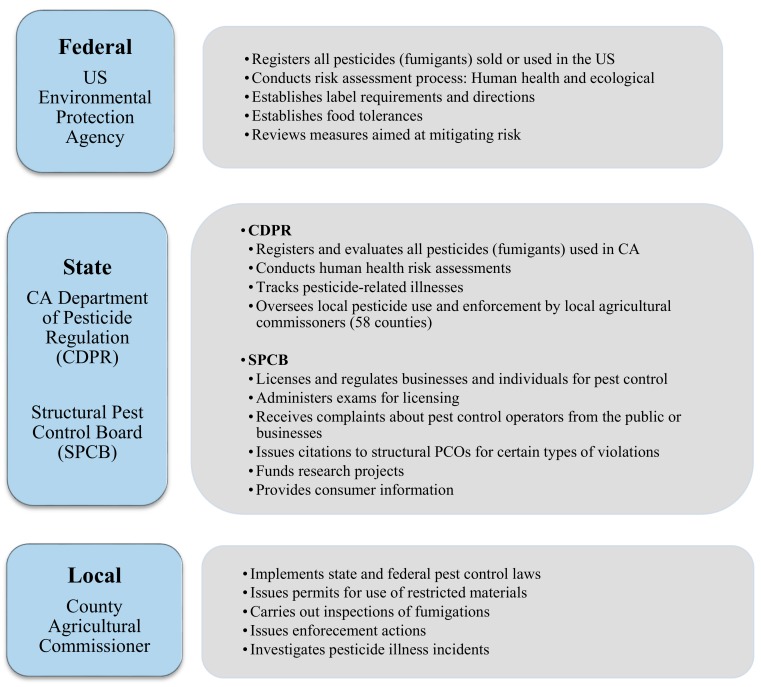
Overview of federal, state, and local agency roles in regulating structural fumigation in California.

In California, pesticide use is regulated and overseen at the federal, state, and local levels, by the US Environmental Protection Agency (USEPA), the CDPR and the California Structural Pest Control Board (SPCB), and the County Agricultural Commissioners, respectively (Figure 3). 

The County Agricultural Commissioner inspects structural fumigations. In California, approximately 2–4% of structural fumigations are inspected [27,28]. An inspection can occur at any stage of the fumigation process; there are no requirements for the inspector to be onsite for the clearance/certification phase. The Agricultural Commissioner’s office does not conduct air testing of fumigated structures and relies on the SPCO for this. 

SPCOs must be licensed and receive training to ensure safe handling and use of pesticides and proper use of safety equipment. Distributors of sulfuryl fluoride gas require a product stewardship training program for all pest control companies that use their fumigant. However, in December 2016, the USEPA Office of the Inspector General issued a report titled, “Additional Measures Can Be Taken to Prevent Deaths and Serious Injuries From Residential Fumigations.” The report noted that state pesticide agencies did not have controls in place to ensure that pest control companies attended the required training [29]. 

## 8. Efficacy and Accuracy of Monitoring 

SPCOs are required to follow the directions and precautions listed on the manufacturer’s label. Sulfuryl fluoride is marketed in the United States under four brand names: Vikane, ProFume, Master Fume (Douglas Products), and Zythor (Ensystex). There are three industry-approved sulfuryl fluoride detection devices for measuring residual sulfuryl fluoride in air: The Interscan, Spectros ExplorIR, and the Miran SapphIRe gas analyzers [22]. As of 2016, the USEPA had not evaluated the effectiveness of clearance devices, and according to the report mentioned above by the Office of the Inspector General, USEPA “relies on self-regulation by the device industry” [29]. In the case of the Florida boy with permanent neurological damage, the SPCO lacked a functioning clearance device [12].

Having properly maintained and calibrated monitoring equipment is paramount for ensuring accurate results and the health and safety of the residents following structural fumigation. However, equipment efficacy is not regulated or overseen. The USEPA Office of the Inspector General found that “ineffective devices” are used to certify houses for reentry. The USEPA lacks assurance as to whether the instruments listed by industry on the fumigant label are effective at measuring sulfuryl fluoride at the mandated clearance levels for reentry and whether SPCOs that use these instruments are adequately trained [29]. 

When asked for the calibration records of the monitoring equipment, the SPCO did not have calibration records for the equipment used to clear the residence described in this case report, so it is not possible to know if the equipment was working accurately at the time [30]. The SPCO did provide records indicating they had sent their equipment in for calibration ten days after the incident occurred [30]. There are no regulations in California requiring SPCOs to maintain calibration records. 

Considering the thousands of fumigations performed every year and the limited resources of local jurisdictions, inspecting every fumigation at the clearance phase is not feasible. However, technology is available that could allow the regulatory agencies to receive clearance measurements with geographic information system (GIS) coordinates from all fumigations in real time, using M2M (machine-to-machine) communication and a cloud platform. M2M communication is often used for remote monitoring, which uses an internet-connected network of computing devices and sensors embedded in everyday objects to transmit data. This technology could provide local jurisdiction inspectors the ability to verify the certification phase in real time, thus increasing the number of reviews of structural fumigation from 2–4% to nearly 100% (i.e., full inspections of every phase would remain infeasible). Increasing oversight and having the ability to verify clearance testing (e.g., number of tests, time, location, and results) could also increase the accountability of SPCOs and could help inspectors identify gaps in clearance testing. It would also provide valuable exposure information in situations where illnesses are reported.

## 9. Conclusions

The physical and toxicological characteristics of sulfuryl fluoride, in combination with limitations in regulatory oversight, appear to be underlying factors contributing to continued exposure and illnesses seen after structural fumigation in California and other states. The current clearance level (1 ppm) and sensitivity limitations with direct reading instruments are barriers for understanding whether exposures post-clearance exceed acute and/or sub-chronic RfCs. Using TWAs alone for measuring sulfuryl fluoride exposure dilutes and masks acute exposure concentrations relevant for understanding health impacts. Recent advances in technology and improvements in regulation could help mitigate issues such as residual sulfuryl fluoride releases from structural voids, desorption from various materials, inadequate aeration times, lack of oversight of clearance equipment, verification of mandatory SPCO training, and limited inspections of structural fumigations. 

In light of continued exposures and data regarding sulfuryl fluoride’s characteristics, the following measures could be considered: (1) increasing aeration times before clearance; (2) ensuring that literature provided to consumers includes information on all household commodities (e.g., mattresses with air bladders, diving buoyancy compensator devices (BCDs), inflatable toys, and neoprene wetsuits) and structural voids (e.g., wall sockets, cabinets, and crawl spaces) that can trap sulfuryl fluoride, and steps for mitigation; (3) creating mechanisms for regulatory oversight of clearance devices to address efficacy and maintenance; (4) implementing available technology and increasing inspections at the clearance phase of structural fumigation; and (5) requiring data collection, storage, and verification of clearance levels measured by the SPCO. These changes could reduce exposures and illness caused by sulfuryl fluoride following structural fumigation.

## Figures and Tables

**Table 1:** Summary of physical characteristics and toxicological effects of sulfuryl fluoride and chloropicrin.

**Sulfuryl Fluoride**	**Chloropicrin**
**Physical Characteristics at Room Temperature**	
• odorless • colorless • **gas** • **non-reactive** • volatile	• **sharp highly irritating odor** • colorless to faint yellow • **liquid** * • **reactive** • volatile
**Toxicological Effects (Acute)**	
• central nervous system depressant • nausea • **vomiting** • abdominal pain • respiratory irritant • coughing • chest tightness • headache • **slurred speech** • **itching** • **seizures** • **muscle twitching** • **restlessness** • **pulmonary edema**	• **lachrymator** • **burning eyes** • nausea • headache • respiratory irritant • difficulty breathing • coughing

Note: Some characteristics of chloropicrin may vary depending on use; those stated above are reflective of use as a structural fumigation warning agent. Characteristics listed in boldface are those considered unique to the compound as applied in its intended use for structural fumigation [3,4]. * Liquid form when used as a structural fumigation warning agent.

**Table 2:** Examples of incidents involving sulfuryl fluoride used in structural fumigation, from the Pesticide Illness Surveillance Data, California Department of Pesticide Regulation [11].

Reported sulfuryl fluoride incidents involving residents or others post-clearance		
Year, Case # and County	Medical Description	Narrative Description
2003-9 Ventura	Palpitations, breathing difficulty, inability to concentrate. Breathing difficulties. Her husband reported her to having panic attacks reliving this incident.	An SPCO fumigated about ten condominiums. He returned two days later, removed the tarps and cleared them for reentry the next morning. A couple returned to their condo late that afternoon but developed symptoms 12 hours later. The couple slept in their bed that night. They awoke early and could not breathe so they left the apartment for fresh air and felt better. **The SPCO tested the apartment and found the mattress to be off-gassing the fumigant.** *
2003-293 Los Angeles	Itchy throat, shortness of breath, cough.	An SPCO fumigated some townhouses in a condominium complex. After the SPCO cleared the condominiums for reentry, a 12-year-old asthmatic boy reentered his condominium. He began suffering respiratory problems and sought medical attention.
2007-114 San Diego	Time of exposure: Lightheadedness, headache; several days later: Intermittent tingling of the right hand and right foot.	A utility technician experienced symptoms after turning on the gas at a recently fumigated home. **Tests revealed levels between 0 and 4 ppm sulfuryl fluoride 12 days after the SPCO cleared the home.** The residents smelled an odor and reported similar symptoms.
**Incidents involving banks**		
2003-632 Alameda	Watery eyes, runny nose, sore throat, nausea, slight headache, slight cough, metallic taste in the mouth.	A bank employee assisted the SPCO in clearing the bank vault following fumigation. **Accounts differ as to whether she entered the vault that retained 20 ppm fumigant.** Either the fumigant or chloropicrin warning agent could have caused her symptoms.
2008-1063 Contra Costa	Irritation of the eyes and throat, mild coughing.	The day after fumigators cleared the building, two bank tellers entered a small room by the vault. They developed irritant symptoms that resolved quickly in fresh air. The human resources director had them taken for evaluation at a clinic. They were referred to a hospital where staff consulted poison control and received reassurance on safety in pregnancy. The SPCO re-checked the area and found it safe. The warning agent is a more likely cause of the symptoms than the fumigant.
2010-813 Monterey	Nausea, vomiting, lightheaded. She told her manager that she felt like she was going to black out. A co-worker said that she had vomited but she did not mention this during her interview. Felt congested, throat discomfort, migraine. The next day, she said she had a headache and felt like she “had a cold.” Developed a bad taste in his mouth within 15 minutes of working. Burning eyes, headache, nausea, vomiting. Headache began within 15 minutes of starting to work, followed by burning and irritated eyes (she wore contact lens) Headache, difficulty breathing, migraine, lightheaded, dizziness. He noticed a rotten egg smell when he arrived to open the bank. Diagnosis: headache, bronchospasm, adverse reaction to insecticide. Headache, blurred vision, nausea, numbness in extremities, funny taste in mouth. The chemical smell made her feel thirsty. She said she had a history of migraines and anxiety.	Eight bank employees reported symptoms when they returned to work the following Monday morning after their workplace had been fumigated with sulfuryl fluoride over the weekend. Before bank employees arrived, the SPCO checked levels of sulfuryl fluoride inside the bank, which were negative. The vault had not been aerated over the weekend because staff needed to be present. **Readings inside were 2–3 ppm.** Fans were placed inside the vault to aerate it for about 45 minutes, then the bank was certified for reentry. Within 15 minutes of work, employees began feeling ill. One customer left because of the fumes. The assistant manager called the environmental health department when several employees and a customer complained. The hazmat specialist evacuated the bank. When the SPCO arrived, he took readings for the entire building. Label requires readings be taken in the breathing zones of the fumigated area. The hazmat specialist asked for additional readings to be taken near the floor of the main area and vault. Tests were positive in both areas. **Possible sources for the rising levels may have been from sulfuryl fluoride seeping out of locked drawers and cabinets that were not aerated.** The SPCO aerated the bank again until no sulfuryl fluoride was detected.

* Emphasis added (bold).

## References

[1] California Office of Emergency Services (2016). Hazardous Materials Spill Report: CalOES Control #16-1288.

[2] Orange County Agricultural Commissioner (2016). Pesticide Episode Investigation Report: WHS No. 10-ORA-16.

[3] California Department of Pesticide Regulation Sulfuryl Fluoride Risk Characterization Document, Volume I: Health Risk Assessment. Medical Toxicology Branch, 2006a California Environmental Protection Agency. http://www.cdpr.ca.gov/docs/emon/pubs/tac/tacpdfs/sulfluor/final_rcd_vol1.pdf.

[4] National Pesticide Information Center Sulfuryl Fluoride Technical Fact Sheet. Updated 2011. http://npic.orst.edu/factsheets/archive/sftech.html.

[5] California Department of Pesticide Regulation (2013). Structural Fumigation—California Aeration Plan. California Environmental Protection Agency. http://www.cdpr.ca.gov/docs/dept/prec/2013/071913_aeration_plan.pdf.

[6] US Government Publishing Office (2017). Electronic Code of Federal Regulations—Tolerances and Exemptions for Pesticide Chemical Residues in Food (Title 40, Chapter I, Subchapter E, Part 180). https://www.ecfr.gov/cgi-bin/text-idx?SID=a3b649316ccb17c31211db2edd81f789&mc=true&node=pt40.24.180&rgn=div5#se40.26.180_1575.

[7] US Environmental Protection Agency About Pesticide Tolerances. Updated September 2016. https://www.epa.gov/pesticide-tolerances/about-pesticide-tolerances.

[8] California Department of Pesticide Regulation (2016). Results from Post-Fumigation Monitoring of a Structure Associated with a Pesticide Illness Report from Orange County.

[9] University of California Berkeley Structural Fumigation. https://nature.berkeley.edu/upmc/fumigation.php.

[10] Structural Pest Control Board (2013). Questions and Answers about Fumigation. California Department of Consumer Affairs. http://www.pestboard.ca.gov/forms/fumigate.pdf.

[11] California Department of Pesticide Regulation California Pesticide Illness Query 2003–2014. California Environmental Protection Agency. http://apps.cdpr.ca.gov/calpiq/.

[12] Centers for Disease Control Notes from the Field: Acute Sulfuryl Fluoride Poisoning in a Family—Florida, August 2015. U.S. Department of Health and Human Services. https://www.cdc.gov/mmwr/volumes/65/wr/mm6527a4.htm.

[13] California Poison Control System (2018). Calls to Poison Control System Regarding Sulfuryl Fluoride Exposure Concerns 2010–2016.

[14] California Department of Pesticide Regulation (2006). Sulfuryl Fluoride Risk Characterization Document; Volume II. Medical Toxicology Branch, California Environmental Protection Agency. http://www.cdpr.ca.gov/docs/emon/pubs/tac/tacpdfs/sulfluor/final_rcd_vol2.pdf.

[15] National Library of Medicine Sulfuryl Fluoride. Toxnet. Last Updated 2007. https://toxnet.nlm.nih.gov/cgi-bin/sis/search/a?dbs+hsdb:@term+@DOCNO+828.

[16] National Pesticide Information Center Pesticides and Children. Last Updated 2015. http://npic.orst.edu/health/child.html.

[17] US Environmental Protection Agency Acute Exposure Guideline Levels-Sulfuryl Fluoride 2699-79-8. Last Updated 2016. https://www.epa.gov/aegl/sulfuryl-fluoride-results-aegl-program.

[18] US Environmental Protection Agency (2002). A Review of the Reference Dose and Reference Concentration Processes. https://www.epa.gov/sites/production/files/2014-12/documents/rfd-final.pdf.

[19] Office of the Federal Register (2011). A Guide to the Rulemaking Process. https://www.federalregister.gov/uploads/2011/01/the_rulemaking_process.pdf.

[20] California Department of Pesticide Regulation (2017). Establishing Sulfuryl Fluoride Uncertainty Factors for Acute and Short-Term Exposures. http://www.cdpr.ca.gov/docs/risk/rcd/establishing_sulfuryl_fluoride.pdf.

[21] Mühle J., Huang J., Weiss R.F., Prinn R.G., Miller B.R., Salameh P.K., Harth C.M., Fraser P.J., Porter L.W., Greally B.R. (2009). Sulfuryl fluoride in the global atmosphere. J. Geophys. Res. Atmos..

[22] Douglas Products Vikane Fact Sheet. https://vikanefumigant.com/wp-content/uploads/2017/09/1_U01-069-146_Fact-Sheet-ENGLISH_HR.pdf.

[23] California Department of Public Health Pesticide Hazard Alert—Workers Became Ill after Bank Was Fumigated. Occupational Health Branch. https://www.cdph.ca.gov/Programs/CCDPHP/DEODC/OHB/OPIPP/CDPH%20Document%20Library/PestAlertBank1.pdf.

[24] Scheffrahn R. (1987). Desorption of residual sulfuryl fluoride from structural and household commodities by headspace analysis using gas chromatography. Environ. Contam. Toxicol..

[25] Scheffrahn R. (1992). Indoor airborne residues of methly bromide and sulfuryl fluoride following aeration of fumigated houses. Indoor Air.

[26] Shurdut B. (1995). Amended report for evaluation of concentrations of sulfuryl fluoride inside houses following fumigation with VIKANE gas fumigant. Indianapolis Indiana: Global Human Exposure Assessment.

[27] San Diego County Agricultural Commissioner Pesticide Regulation Program Work Plan 2015–2016. http://apps.cdpr.ca.gov/docs/county/workplan/CY2015/san_diego_plan.pdf.

[28] Orange County Agricultural Commissioner Pesticide Use Enforcement Work Plan 2015–2017. http://apps.cdpr.ca.gov/docs/county/workplan/CY2016/orange_plan.pdf.

[29] U.S. Environmental Protection Agency (2016). Additional Measures Can Be Taken to Prevent Deaths and Serious Injuries from Residential Fumigations.

[30] Trumbauer D. (2016). Letter from Damara Trumbauer, Orange County Agricultural Commissioner to Tracy Barreau.

